# Endoscopic Ultrasound for the Management of Pancreatic Neuroendocrine Tumors: Diagnosis, Treatment, and Future Perspectives

**DOI:** 10.1111/jgh.70302

**Published:** 2026-02-26

**Authors:** Angelo Bruni, David Meacci, Roberto Falbo, Marco Fichera, Francesco Poggioli, Riccardo Casadei, Claudio Ricci, Leonardo Henry Eusebi

**Affiliations:** ^1^ Gastroenterology Unit IRCCS Azienda Ospedaliero‐Universitaria di Bologna Bologna Italy; ^2^ Department of Medical and Surgical Sciences University of Bologna Bologna Italy; ^3^ Pancreatic Surgery Unit IRCCS Azienda Ospedaliero‐Universitaria Di Bologna Bologna Italy

**Keywords:** biliary complications, endoscopic treatment, liver transplant

## Abstract

Pancreatic neuroendocrine tumors (PanNETs) are increasingly diagnosed, reflecting greater clinical awareness, improved imaging, and revised classification. This review summarizes evidence on epidemiology, diagnostic workup, and endoscopic ultrasound (EUS)–guided management of PanNETs, encompassing diagnostic evaluation, tissue acquisition, and therapeutic interventions. EUS provides the highest diagnostic yield for lesions < 20 mm and enables detailed morphologic and vascular assessment through contrast‐enhanced and harmonic EUS, quantitative elastography, and, in selected cases, needle‐based confocal laser endomicroscopy. Fine‐needle biopsy is superior to fine‐needle aspiration for specimen adequacy, immunohistochemistry, and Ki‐67 assessment, but preoperative grading may still be underestimated, with direct impact on decisions between surveillance, surgery, and systemic therapy. Therapeutically, EUS‐guided radiofrequency ablation achieves high short‐term to midterm clinical and radiologic control in insulinomas ≤ 2 cm and in selected small nonfunctioning PanNETs, at the cost of adverse event rates around 15%–20%. Ethanol and microwave ablation are feasible but supported by less mature data, and all EUS‐guided ablation strategies should currently be regarded as investigational, pending long‐term comparative studies versus surgery and active surveillance. In borderline or locally advanced disease, neoadjuvant [^177^Lu]‐Lu‐DOTATATE appears to facilitate resection, but its optimal role remains to be defined. Emerging artificial intelligence and radiomics models using EUS images may refine risk stratification and support a precision endoscopy paradigm in PanNETs.

## 1 Introduction

Neuroendocrine tumors (NETs), along with neuroendocrine carcinomas (NECs), constitute a rare class of malignancies collectively referred to as neuroendocrine neoplasms (NENs) [1]. These tumors arise from cells of the diffuse endocrine system and exhibit distinct biological profiles, contributing to their complex classification and management [2].

Within this field, pancreatic neuroendocrine tumors (PanNETs) represent a paradigmatic case in which advances in endoscopic ultrasound (EUS) have profoundly modified diagnostic pathways, risk stratification, and therapeutic options. The rapid evolution of EUS‐based imaging, tissue acquisition, and locoregional ablative techniques has generated a heterogeneous body of evidence, ranging from small case series to prospective cohorts, often with variable endpoints and follow‐up. As a result, clinicians are increasingly confronted with the challenge of integrating international guideline recommendations, surgical standards, and emerging EUS‐guided therapies in the decision‐making process.

The aim of the present review is to critically evaluate the role of EUS in the diagnosis, staging, and treatment of PanNETs and to outline current and future perspectives for its integration within multidisciplinary management algorithms. Particular emphasis has been placed on the interaction between EUS, somatostatin receptor (SSTR)–based imaging, and evolving histomolecular classifications, given their combined impact on therapeutic personalization.

## 2 Literature Search

In order to identify the most relevant literature for this review and to minimize selection bias, a structured literature search was performed using a comprehensive query of MEDLINE/PubMed, Embase, and the Cochrane Library covering the period from 2000 to October 2025. Only full‐text articles published in English were included, with no restrictions on study design, and study selection was performed independently by two investigators, with any discrepancies resolved by consensus. A recursive search of the reference lists of all eligible articles was also performed to identify additional pertinent studies. A summary of the search strategy is reported in Table 1.

**TABLE 1:** Search strategy summary.

Items	Specification
Date of search	March 31, 2025
Databases and other sources searched	MEDLINE/PubMed, Embase, Cochrane Library
Search terms used (including MeSH and free text search terms and filters)	Combinations of MeSH and free‐text terms for “pancreatic neuroendocrine tumor,” “pancreatic neuroendocrine neoplasm,” “PanNET,” “PanNEN,” “neuroendocrine carcinoma,” “NET,” “NEC,” “endoscopic ultrasound,” “EUS,” “EUS‐FNA,” “EUS‐FNB,” “fine‐needle aspiration,” “fine‐needle biopsy,” “radiofrequency ablation,” “RFA,” “ethanol ablation,” “alcohol ablation,” “microwave ablation,” “surgery,” “pancreatic resection,” “active surveillance”
Timeframe	2000–2025
Inclusion and exclusion criteria (study type, language restrictions etc.)	Inclusion criteria: all study types (randomized and nonrandomized trials, prospective and retrospective observational studies, registries, large case series), full‐text articles, adult patients with PanNET, English language Exclusion criteria: case reports or small case series with fewer than 10 patients for the assessment of therapeutic efficacy, studies not reporting PanNET‐specific outcomes
Selection process	Titles and abstracts of all retrieved records were screened independently by two reviewers (B.A., M.D.). Disagreements were resolved with involvement of a third senior reviewer (E.L.H.) when needed.

## 3 Epidemiology and Clinical Features of PanNETs

PanNETs originate from the islet cells of the pancreas and represent the most prevalent subset of NENs. Their incidence has been steadily increasing, currently estimated at 1/100,000 individuals per year [3]. Gastroenteropancreatic NENs (GEP‐NENs) are now recognized as the second most common malignancy of the gastrointestinal tract, surpassed only by colorectal adenocarcinoma in prevalence [4]. PanNETs account for approximately 6.8% of all primary pancreatic tumors, with a notable 3.9‐fold increase in diagnosed cases [5]. Up to 27% of patients present with metastatic disease at diagnosis, and advanced‐stage PanNETs are associated with a poor prognosis [6]. This rising incidence is likely attributable to increased clinical awareness and advancements in diagnostic imaging technologies in recent years [7].

Epidemiological data reveal a male predominance in countries such as France, the United States, and Norway, whereas, in Italy, PanNETs are more frequently diagnosed in females. The US SEER (Surveillance, Epidemiology, and End Results) program showed that incidence rates were higher among white and African American individuals (0.32 and 0.36 per 100 000, respectively) compared to Asian Americans and American Indians [8].

PanNETs can occur sporadically or in association with hereditary syndromes, including multiple endocrine neoplasia type 1 (MEN1), Von Hippel–Lindau disease, and neurofibromatosis type 1 (NF1) [7]. Key risk factors include advanced age and genetic predisposition (ranging from single‐nucleotide variants to broader hereditary predisposition) [9] age, tumor grade, disease stage, and functional status are key prognostic factors influencing survival in patients with PanNETs [10]. Despite these established associations, the precise pathogenesis of PanNETs remains incompletely understood, underscoring the need for further research into their molecular and environmental determinants.

### 3.1 Classification and Grading Systems

The European Neuroendocrine Tumor Society (ENETS) played a pivotal role in the first structured attempt to classify and stage GEP‐NENs. Recognizing the heterogeneous nature of these neoplasms, ENETS organized its first Consensus Conference in 2006 to establish guidelines aimed at standardizing the stratification and treatment of GEP‐NET patients. This initiative led to the development of the TNM staging system for foregut NETs, which categorized tumors based on their anatomical location (gastric, duodenal, pancreatic), rather than their functional activity, cell type, or genetic background. Additionally, ENETS introduced a grading system, inspired by the World Health Organization (WHO) classification for pulmonary endocrine tumors, based solely on proliferation status, differentiating NETs into G1, G2, and G3 according to mitotic count and Ki‐67 index. This classification aimed to bridge the gap between existing WHO classifications and the need for a more precise prognostic assessment. Although this system represented a major advancement, ENETS acknowledged the need for future validation through clinicopathological studies to further refine NET classification and staging [11]. Since then, the classification of pancreatic NENs has seen considerable progress with the cooperation of the WHO.

In 2010, WHO introduced a grading system for GEP‐NENs [12], stratifying tumors based on Ki‐67 proliferation index and mitotic count: Ki‐67 is a nuclear protein involved in cell cycle regulation expressed throughout all phases of cell duplication; the Ki‐67 index is now determined by assessing at least 500 cells in the most intensely labeled areas. The mitotic index is evaluated by counting mitotic figures in 50 high‐power fields (HPF) within the regions of highest mitotic density and is expressed as mitoses per 10 HPF [13]. Three categories were created: well‐differentiated PanNENs G1 and G2, classified as PanNETs, and poorly differentiated PanNEN G3, classified as pancreatic neuroendocrine carcinomas (PanNECs): PanNETs and PanNECs were considered to be on a single biological spectrum because both entities shared similar features such as the expression of neuroendocrine markers (synaptophysin and chromogranin). PanNET G1 tumors were defined by < 2 mitoses per 10 HPF and Ki‐67 < 2%, whereas PanNET G2 included tumors with 2–20 mitoses per 10 HPF and Ki‐67 between 2% and 20%. PanNECs were classified as G3, characterized by > 20 mitoses per 10 HPF and Ki‐67 > 20%, reflecting their aggressive nature [12]. However, advances in genetic and molecular research revealed significant heterogeneity within the G3 category, leading to the recognition of two distinct subtypes: well‐differentiated PanNETs G3 and poorly differentiated PanNECs, differing in morphology, biological behavior, and therapeutic response.

The 2017 WHO revised NENs' classification formally distinguished PanNETs G3 from PanNECs and adjusted the Ki‐67 cutoff for G1 and G2 from 2% to 3% [13]. The latest 2022 WHO edition of digestive system tumor classification has reinforced this distinction, applying it to all NENs of the digestive tract, emphasizing the biological and clinical differences between high‐grade NETs and NECs as separate biological entities with different precursor cells and distinct molecular biology based on pathological, clinical, and molecular data [13, 14, 15].

The American Joint Committee on Cancer (AJCC) classification for GEP‐NETs has undergone continuous revisions to align with the evolving WHO and the complementary ENETS classifications. Initially, AJCC staged PanNETs using the same system as exocrine pancreatic carcinomas, which proved inadequate due to discrepancies in survival prediction. The eighth edition introduced a separate staging system for PanNETs, PanNECs, and mixed neuroendocrine–nonneuroendocrine neoplasms (MiNENs), incorporating key WHO updates such as the distinction between PanNETs G3 and PanNECs [16]. The most recent AJCC ninth edition further integrates advances in diagnostic imaging and molecular profiling, emphasizing the growing role of endoscopy and SSTR–based imaging in NET management. Additionally, it recognizes emerging prognostic markers, including alternative lengthening of telomeres and ATRX/DAXX loss, which have been associated with increased metastatic potential. Despite these refinements, T, N, and M categories remain unchanged in the latest edition, underscoring the need for further updates to optimize the staging of rare subtypes such as PanNECs and MiNENs [17].

### 3.2 Rare Subtypes

NECs are characterized by inherited high‐grade G3 malignancy, aggressive by definition [1]. A significant diagnostic challenge remains distinguishing PanNETs G3 from PanNECs due to overlapping histological features. Although well‐differentiated PanNETs typically display organoid architectural patterns, “salt‐and‐pepper chromatin,” and uniform ovoid nuclei, PanNETs G3 can exhibit larger nests, sheets, and increased nuclear atypia, making them difficult to differentiate from PanNECs [18]. Oppositely, PanNECs, which share histological similarities with pancreatic ductal adenocarcinomas (PDACs), can be classified into small cell and large cell variants, with small cell NECs featuring a high nuclear‐to‐cytoplasmic ratio, nuclear molding, and dark chromatin, whereas large cell NECs display pleomorphic nuclei, prominent nucleoli, and syncytial aggregates [19]. Despite these distinctions, morphological overlap between PanNETs G3 and PanNECs remains a major limitation in routine pathology.

Additionally, a distinct category known as MiNENs has identified, which exhibits a combination of neuroendocrine and nonneuroendocrine components, typically with poor differentiation. The neuroendocrine component of MiNENs generally demonstrates proliferation indices comparable to other NECs. When feasible, each component should be graded separately to ensure accurate characterization and prognosis [20].

### 3.3 Clinical Features

PanNETs are further classified into two groups based on the secretion of biologically active peptides: functional and nonfunctional tumors [3]. Functional PanNETs secrete hormones that cause distinct clinical syndromes, contributing to the overall disease burden [6]. These syndromes are characterized by specific clinical presentations associated with elevated hormone levels detected in biochemical analyses [21].

Among the most common functional PanNETs, insulinomas induce fasting hypoglycemia due to excessive insulin secretion, whereas gastrinomas are associated with Zollinger–Ellison syndrome, characterized by severe ulcers and acid hypersecretion. Glucagonomas present with a classic triad of diabetes mellitus, necrolytic migratory erythema, and weight loss. VIPomas (Verner–Morrison syndrome) cause secretory diarrhea and electrolyte imbalances [3].

However, the majority of PanNETs (between 50% and 85%) are nonfunctional (NF‐PanNETs) and do not cause hormone‐related clinical syndromes. Although typically indolent, NF‐PanNETs have a higher propensity for late‐stage presentation, with increased tumor burden (T‐stage), lymph node involvement, and liver metastases, leading to worse overall survival outcomes compared to their functional counterparts [22].

The diagnostic landscape of PanNENs has significantly evolved primarily due to the widespread adoption of EUS and other advanced imaging modalities [23]. EUS enables high‐resolution imaging and allows tissue acquisition when necessary; it also demonstrates superior sensitivity in detecting PanNENs compared to other available imaging modalities, resulting in an indispensable tool for PanNETs evaluation [24].

This review aims to further explore the pivotal role of EUS in the management of PanNETs, examining the current state of research on diagnosis and therapy, while outlining future perspectives.

## 4 Diagnostic Role of EUS in PanNETs

EUS is an endoscopic procedure that integrates a mechanical ultrasound transducer and probe at the tip of the endoscope, enabling effective detection of PanNETs due to the anatomical proximity of the pancreas to the stomach and the duodenum. The accuracy of EUS in identifying PanNETs is high, showing a sensitivity ranging from 87% to 93% and a specificity reaching up to 95%–98% [25, 26]. The key technical advantages and clinical implications of EUS‐based diagnostic modalities are summarized in Table 2.

**TABLE 2:** Diagnostic practical recommendations for EUS in PanNETs.

Diagnostic application	Clinical evidence	Practical implication
EUS (B‐mode)	Sensitivity 87%–93%, specificity 95%–98% for PanNETs; detects lesions 2–5 mm, superior to CT (64%–82%) and MRI (74%–100%) for lesions < 20 mm. Essential for insulinomas with low somatostatin receptor expression	First‐choice imaging modality for suspected PanNETs, especially < 20 mm; ideal for early diagnosis and preoperative staging; enables high‐resolution detection of small, functionally active or nonfunctional tumors
CH‐EUS	Provides nearly 100% specificity in distinguishing hypervascular PanNETs from PDAC or inflammatory lesions. Enhanced vascular imaging and tumor grading by vascular phases; hypoenhancement in late phase indicates aggressiveness (sensitivity 86%, specificity 96%)	Useful for assessing tumor biology and prognosis; hypoenhancement signals poor outcome and supports risk stratification
EUS–elastography	Qualitative elastography: blue homogenous pattern common but nonspecific. Quantitative (strain ratio) elastography showed up to 100% sensitivity and 88% specificity versus PDAC	Adjunct tool for differential diagnosis; promising for PanNET vs. PDAC distinction
EUS‐guided FNB vs. FNA for histological diagnosis	FNB yields higher diagnostic adequacy (up to 95%) and better Ki‐67 index assessment compared to FNA; larger, preserved core samples improve tumor grading and classification	Preferred method for preoperative histological confirmation and grading; essential for identifying tumor proliferation rate and guiding management decisions; critical for distinguishing G1 vs. G2 tumors, which impacts surveillance vs. intervention strategies
n‐CLE	Alone: 70% accuracy (vs. 90% FNA, 95% FNB); diagnostic in 50% of FNA‐inconclusive cases. Combined with FNA: 96.7% accuracy	Alone: 70% accuracy (vs. 90% FNA, 95% FNB); diagnostic in 50% of FNA‐inconclusive cases. Combined with FNA: 96.7% accuracy
EUS for surveillance of small low‐grade PanNETs	Surveillance supported for asymptomatic, well‐differentiated NF‐PanNETs < 2 cm, particularly G1 (Ki‐67 ≤ 2%); EUS allows accurate measurement of growth rate and vascular changes; studies show low progression rate (1%–10%) during follow‐up	MPD involvement, or change in vascularity indicating malignant transformation or the need for therapeutic escalation

Abbreviations: CE‐EUS = contrast‐enhanced endoscopic ultrasound, EUS = endoscopic ultrasound, FNA = fine‐needle aspiration, FNB = fine‐needle biopsy, MPD = main pancreatic duct, n‐CLE = needle‐based confocal laser endomicroscopy, NF = nonfunctioning, PanNET(s) = pancreatic neuroendocrine tumor(s), PDAC = pancreatic ductal adenocarcinoma.

Computer tomography (CT) and magnetic resonance imaging (MRI) demonstrate good diagnostic performance for lesions greater than 20 mm [27], with sensitivity ranging from 64% to 82% for CT and from 74% to 100% for MRI. However, their ability to detect smaller tumors is limited, and lesions as small as 2–5 mm may be overlooked [28, 29]. In this context, EUS plays a pivotal role, providing superior detection rates for small (< 20 mm) PanNETs. A multicenter study demonstrated that EUS outperforms CT across all lesion sizes, with a pronounced advantage for tumors ≤ 10 mm and particularly those ≤ 5 mm [30]. This advantage is especially relevant for functioning tumors, such as insulinomas, which often remain undetected by CT, MRI, and positron emission tomography (PET). Advanced modalities such as detective flow imaging (DFI‐EUS) can further improve detection through visualization of subtle vascular patterns [31]. Furthermore, EUS is crucial for the detection of multifocal disease in hereditary syndromes such as MEN1 [32]: in these hereditary settings, a prospective study in MEN1 patients showed that EUS detected the majority of small PanNETs, identifying 86 tumors in 90 patients, thereby supporting its role in early diagnosis and surveillance [33]. Collectively, these findings reinforce EUS as an essential tool for the detection of minute (< 10 mm) and small PanNETs.

**FIGURE 1:**
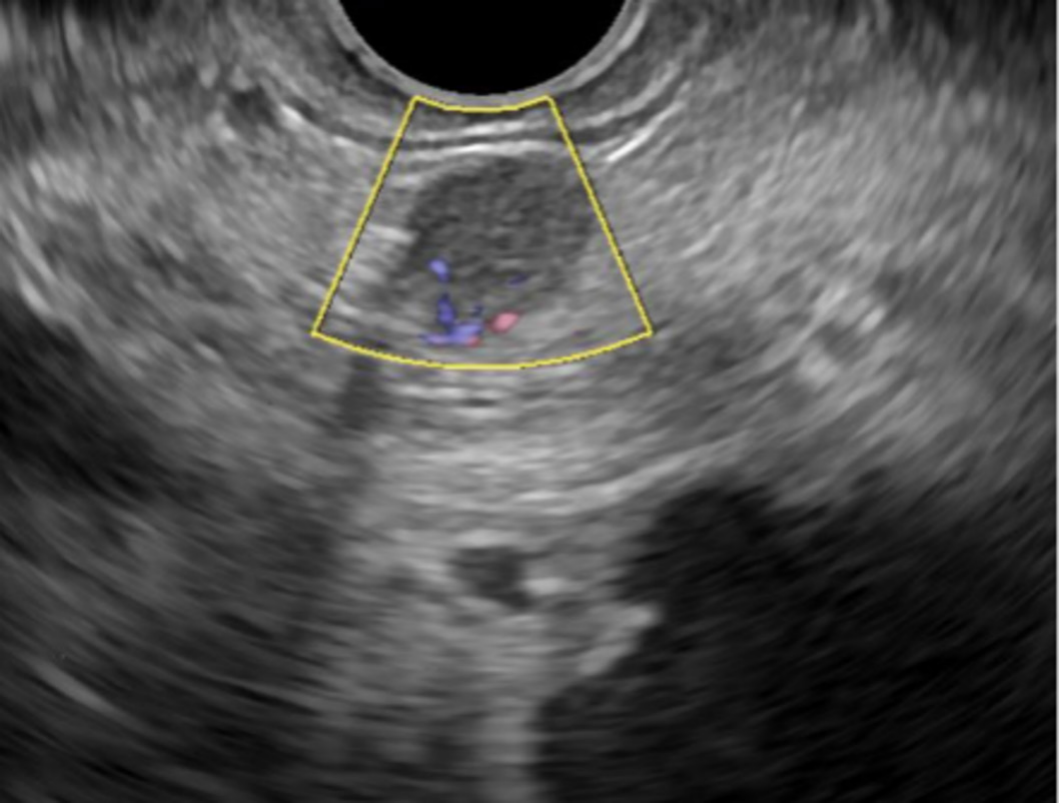
EUS color Doppler view demonstrates a well‐circumscribed hypoechoic nodule in the pancreas showing intralesional bidirectional vascular signals of a small hypervascular, nonfunctioning PanNET.

Furthermore, EUS provides detailed characterization of malignant features, including vascular infiltration, peripancreatic lymph node involvement, and tumor differentiation (Figure 1) [34].

On the other hand, SSTR PET/CT has become a central tool in PanNETs management, serving as the reference imaging modality for initial staging, localization, preoperative assessment, and selection for PRRT, as recognized by SNMMI/EANM Appropriate Use Criteria [35]. A recent Delphi consensus further highlighted that in selected scenarios—surgical candidates with > 2‐cm SSTR‐avid masses, multifocal disease, or functioning tumors—SSTR PET/CT may be used as the initial targeted diagnostic step, sometimes obviating tissue sampling before surgery [36].

Nevertheless, EUS remains an essential diagnostic tool. European guidelines emphasize its role in potentially resectable tumors for evaluating size, vascular and ductal relationships, and for EUS‐guided tissue sampling when it can influence management. It is particularly valuable for small nonfunctioning Pan‐NETs that may be missed on PET/CT and for insulinomas, which often lack significant SSTR expression, achieving high sensitivity and specificity and allowing functional or histological characterization when needed [21, 22, 37]. North American guidelines further recommend EUS–fine‐needle aspiration (FNA)/fine‐needle biopsy (FNB) when imaging is equivocal, tumor grade is uncertain, or multifocality is suspected, advising against its use if it would not alter management [38].

Taken together, SSTR PET/CT and EUS should be considered complementary: PET provides whole‐body functional staging, whereas EUS refines local assessment and enables tissue confirmation when clinically meaningful.

Diagnostic techniques that can be performed via EUS include the standard gray‐scale imaging, elastography, contrast‐enhanced EUS, contrast‐enhanced harmonic EUS (CH‐EUS), and needle‐based confocal laser endomicroscopy (n‐CLE). Moreover, EUS also allows sampling of PanNETs through FNA (EUS‐FNA) and FNB (EUS‐FNB) to achieve cytological and histological diagnosis.

### 4.1 PanNETs Features in EUS

PanNETs exhibit similar characteristics in both transabdominal ultrasonography and EUS; however, EUS offers a superior diagnostic performance as it is not affected by subcutaneous or peritoneal fat. The pancreas can be evaluated through the gastric wall, allowing visualization mainly of the body and the tail, whereas the head and the neck are accessible via the duodenum. The preoperative use of EUS has been shown to increase the detection rate by 25% following a CT scan, highlighting its high sensitivity in tumor identification [39].

PanNETs are typically described as round, homogeneous, hypoechoic, well‐defined lesions [40, 41, 42]. However, these characteristics are not exclusive for PanNETs and must be differentiated from other pancreatic masses, such as serous cystic neoplasms [43], solid pseudopapillary neoplasms [44], intrapancreatic accessory spleen [45], and pancreatic metastasis [46]. In some cases, PanNETs may appear as isoechogenic or hyperechogenic masses with poorly defined margins. Atypical presentations of NETs, particularly in large tumors, are associated with a poorer prognosis because predictive of high‐grade NETs [47]. Indeed, PanNET G3 and PanNEC show similar ultrasonography features with pancreatic acinic cell carcinoma and PDAC [48, 49]. A prospective study by Saizu et al. investigated the most common ultrasonographic features among 165 pancreatic lesions, 24 of which were PanNETs. The key sonographic characteristics linked to a histopathological diagnosis of NET were well‐defined margins (79% vs. 26%, *p* < 0.001), tumor vascularization (67% vs. 25%, *p* < 0.001), hypoechoic rim (46% vs. 10%, *p* < 0.001), and homogenous pattern (46% vs. 9.9%, *p* < 0.001) [34].

Although PanNETs are predominantly solid tumors, some may present as cystic lesions due to the cystic degeneration or calcification resulting from tumor hemorrhage, a phenomenon more commonly observed in low‐grade tumors, whereas a loss of vascular signal on imaging is often associated with poorly differentiated lesions [50].

Additional ultrasound findings suggestive of PanNETs are the intraductal invasion of the main pancreatic duct (MPD), which is more frequently observed in G3 tumors with invasive growth [51], and MPD strictures. Notably, the presence of ductal strictures should not be immediately considered an indicator of malignancy, as they may result from either tumor expansion or serotonin‐induced stromal fibrosis; both of these mechanisms are associated with well‐differentiated PanNETs [24, 52].

### 4.2 Elastography

Elastography is a technique used to assess the elasticity of a specific tissue region. It is classified into two types: qualitative and quantitative. Qualitative elastography evaluates tissue stiffness using a color‐coded pattern. During assessment, PanNETs are typically described as displaying a homogenous blue pattern [53]. However, this characteristic is highly operator‐dependent and lacks specificity, as several non‐PanNET lesions can exhibit similar features [54].

Quantitative elastography has the potential to overcome the intraobserver and interobserver variability associated with qualitative methods. Two notable quantitative approaches include strain ratio elastography, which compares the hardness of a lesion with that of normal pancreatic parenchyma, and shear wave elastography, which measures tissue stiffness independently. A study by Iglesias‐Garcia et al. reported that strain ratio elastography achieved 100% sensitivity and 88% specificity in differentiating PanNETs from PDAC [55]. Despite its promising diagnostic potential, further research is needed to establish the definitive role of EUS–elastography in the detection and characterization of PanNETs.

### 4.3 Contrast‐Enhanced EUS

ce‐EUS is a technique that utilizes a microbubble‐based contrast agent, which is disrupted by ultrasound waves to enhance the signal from highly vascularized lesions. As previously mentioned, PanNETs are well‐vascularized tumors due to the expression of proangiogenic factors [50]. This characteristic causes them to appear hyperenhanced during both early and delayed phases of imaging, whereas PDAC is a fibrotic tumor with reduced vascularization, remaining hypoenhanced after contrast injection [49]. These distinct enhancement patterns improve the differential diagnostic capabilities of EUS for pancreatic lesions, yielding a sensitivity of 92% and specificity near 100% [56].

Beyond differentiating PanNETs from other pancreatic tumors, CE‐EUS features can also provide valuable insights into tumor malignancy. Ishikawa et al. demonstrated that different CE‐EUS enhancement patterns correlate with tumor differentiation grade, confirmed through histological analysis postresection [57]. According to his study, lesions with a diffuse enhancement after contrast injection (Type A) are highly probably benign; instead, filling defects (Type B) may be the expression of necrosis and hemorrhage, features more typical of malignant tumors, whereas no enhancement after contrast (Type C) is strongly associated with a high‐grade neoplasm. When Type B and Type C patterns are considered indicative of malignancy, CE‐EUS archives an accuracy of 90.5%, with a sensitivity of 90% and a specificity of 90% [57].

### 4.4 Contrast‐Enhanced Harmonic EUS

CH‐EUS is an advanced evolution of conventional CE‐EUS, in which the signal generated by microbubbles reflection is filtered to selectively detect harmonic components. This refinement enhances the ability to assess the vascular characteristics of suspicious lesions. Studies have reported that CH‐EUS can differentiate between low‐ and high‐grade tumors by evaluating the vascular pattern with high accuracy, achieving a sensitivity of 86% and a specificity of 96% [58].

Tamura et al. demonstrated that different CH‐EUS enhancement patterns correlate with tumor malignancy, aggressiveness, and prognosis [59]. Their study categorized PanNETs into three groups based on the enhancement during early and late phases: Group A hyperenhanced during both of the phases, Group B hyperenhanced only in the early phase, and Group C hypoenhanced in both phases. Among these, hypoenhanced in the late phase was the most reliable indicator of tumor aggressiveness, with an accuracy of 89.2%, sensitivity of 84.6%, and a specificity of 91.7%. Furthermore, 1‐year survival rates differed significantly among the three groups: 100% in Group A, 60% in Group B, and 43% in Group C (*p* < 0.001). These findings strongly support the predictive value of poor vascularization in the late phase as a marker of high‐grade PanNETs.

### 4.5 Fine‐Needle Aspiration and Fine‐Needle Biopsy

Although EUS features can aid in distinguishing PanNETs from other pancreatic lesions and assessing tumor aggressiveness, definitive diagnosis requires cytological and histological determination. FNA enables the collection of neoplastic specimens through the aspiration of small tissue samples, facilitating differential diagnosis. The highest diagnostic yield is observed in small lesions (< 2 cm), located in the pancreatic tail, with low stromal fibrosis [60, 61].

However, the sensitivity and specificity of FNA for PanNETs diagnosis vary across studies. A recent retrospective analysis reported an 89% diagnostic rate and a 100% concordance in confirmed cases [62]. Another crucial application of FNA is the evaluation of tumor aggressiveness through the Ki‐67 labeling index, which is essential for grading neoplasms. However, obtaining a high‐quality sample with FNA sufficient for determining the Ki‐67 index and grading can be challenging due to poor cellular cohesion, the hypervascular nature of PanNETs, and the limited number of tumor cells typically collected for immunohistochemical staining [63, 64].

FNB represents an evolution of FNA and is increasingly replacing it in clinical practice. Unlike FNA, FNB employs needles with a side fenestrated or end‐cutting design, allowing for the collection of larger tissue samples, which facilitates immunohistochemical analysis and precise Ki‐67 index determination [65, 66].

Several studies have assessed the diagnostic performance of FNA and FNB in pancreatic lesions. A recent retrospective study, designed by Bellocchi et al., compared FNA and FNB in terms of diagnostic adequacy (the proportion of samples suitable for cytohistological evaluation) and diagnostic accuracy in small pancreatic lesions; the final diagnosis was based on either surgical specimen analysis or disease progression after 6 months, with most of the patients diagnosed with a PanNET. The study finding demonstrated the superiority of FNB, which achieved a greater diagnostic accuracy (89.8% vs. 79.1%, *p* = 0.013) and a higher diagnostic adequacy (95.9% vs. 86.1%, *p* < 0.001) [67]. Several meta‐analyses confirmed not only a superior performance of FNB in terms of adequacy and accuracy, but also a comparable complication rate relative to FNA, with fewer needle passes required to obtain a sufficient specimen for FNB [68, 69]. Specifically regarding PanNETs, a retrospective analysis based on a prospective compiled database of patients diagnosed with PanNETs compared sensitivity of FNA and FNB. FNB outperformed FNA in terms of obtaining an adequate sample (85% vs. 77%) and in diagnostic sensitivity among the adequate samples (94% vs. 88%) [70].

However, despite improving PanNETs diagnosis, FNB still has limitations in accurately grading tumors. Several studies indicate that FNB tends to undergrade PanNETs, particularly G2 tumors, potentially leading to undertreatment. A recent study on 53 PanNET patients who underwent FNB followed by surgical resection found that 25% of preoperative G1 and G2 tumor classifications were discordant with postoperative assessment. The most frequent misclassification was G2 underestimation (61% of the overall), whereas G1 tumors were rarely overgraded (6% of the overall). Although FNB is a valuable tool for PanNET diagnosis, its reliability in tumor grading remains uncertain, necessitating further research to optimize its role in clinical management. Underestimation of histological grading can have a significant impact not only on the management of patients eligible for surgical resection, but also on the selection of systemic therapy for patients with locally advanced or metastatic disease [37, 71].

A practical example involves nonfunctional, asymptomatic, well‐differentiated PanNETs of small size (< 20 mm), which, according to current guidelines, may be managed with an approach of active surveillance (“watch and wait”). However, when cytological or histological analysis reveals a Grade 2 or 3 tumor, surgical resection becomes the preferred option, even if the tumor initially appears to be low‐grade [37, 63].

In the case of patients with isolated or predominantly hepatic metastases, surgical resection may be considered if the preoperative Ki‐67 value is ≤ 10%. Conversely, if the Ki‐67 value exceeds this threshold, systemic therapy may become the primary treatment strategy, unless the patient is highly selected and surgical resection is still deemed appropriate [37].

These examples underscore the critical importance of accurate histological grading and preoperative Ki‐67 assessment, as undergrading can significantly alter therapeutic management.

### 4.6 Needle‐Based Confocal Laser Endomicroscopy

Another limitation of FNA and FNB is the difficulty in obtaining sufficient specimen for cytological or histological analysis, particularly for very small lesions. A promising alternative technique is n‐CLE, a technique that may overcome this challenge. n‐CLE utilizes fluorescent dyes and mini‐probes that can pass through an FNA needle, allowing in vivo histological assessment without tissue extraction [72]. A study by Yamada et al. demonstrated that n‐CLE alone has lower diagnostic accuracy than FNA or FNB alone (70%, 90%, and 95%, respectively) [73]. However, n‐CLE resulted diagnostic in 50% of cases in which FNA resulted inconclusive, and when combined with FNA, the overall diagnostic accuracy for PanNETs increased to 96.7%. In the future, integrating n‐CLE with other endoscopic techniques may further enhance the diagnostic performance of EUS, improving early and accurate detection of PanNETs.

## 5 Therapeutic Role of EUS in PanNETs

EUS has progressed from a purely diagnostic modality to a pivotal therapeutic tool in the management of PanNETs. Its therapeutic applications include ablation techniques, such as radiofrequency ablation (RFA), ethanol ablation (EUS‐EA), and other emerging experimental approaches. Evidence increasingly shows that EUS‐guided interventions can provide excellent disease control, often reducing the morbidity associated with pancreatectomy or other surgical procedures [74, 75]. Choosing between observation and resection depends on multiple factors: tumor size, patient age, comorbidities, tumor location, growth dynamics, and patient preference. Because this decision can be complex, it is considered a “gray zone” and should always involve a multidisciplinary team approach before discussion with the patient [22, 37]. Recent findings suggest that a nonsurgical “active observation” strategy with annual clinical and radiological follow‐up is justified for tumors < 2 cm, as indicated by preliminary data from the multicenter ASPEN study [76]. However, living with a tumor that carries a malignant potential can be difficult to accept, particularly for younger patients who would require long‐term imaging surveillance. Table 3 provides a summary of the principal therapeutic guidance for EUS‐based management of PanNETs.

**TABLE 3:** Therapeutic practical recommendations for EUS in PanNETs.

Technique	Mechanism of action	Indications	Key considerations
EUS‐guided RFA	Coagulative necrosis via localized radiofrequency energy	Symptomatic insulinomas or NF‐PanNETs, < 2 cm, well‐differentiated, in patients unfit or unwilling for surgery	‐ High complete ablation rate: ~90%–95% in insulinomas, ~80%–90% in NF‐PanNETs ‐ AE rate: ~10%–20% overall; severe AEs ~3% ‐ Maintain ≥ 2 mm from MPD to reduce pancreatitis risk (≤ 1 mm: up to 40% risk) ‐ Continuous 10% dextrose infusion for insulinomas ‐ Role of NSAIDs and stenting remains controversial
EUS‐guided ethanol ablation	Chemical necrosis by ethanol‐induced cell lysis	Symptomatic insulinomas or NF‐PanNETs, < 2 cm, well‐differentiated, in patients unfit or unwilling for surgery	‐ Moderate complete ablation rate: ~60%–88% of cases ‐ AE rate: 3%–20%, mostly mild; pancreatitis up to 14.6% ‐ Uneven diffusion may lead to partial ablation and recurrence ‐ Multiple sessions may be needed
EUS‐guided microwave ablation	Coagulative necrosis induced by electromagnetic microwave energy	Symptomatic insulinomas or NF. PanNETs in patients unfit or unwilling for surgery, poorly responsive to ethanol or RFA	‐ Small case series report 100% technical success ‐ AE rates variable (3%–36%), mainly mild ‐ Delivers uniform thermal necrosis ‐ Limited data on optimal settings, long‐term efficacy, and recurrence rates

*Note:* Clinical success defined as complete tumor ablation on imaging for NF‐PanNETs or symptoms resolution for F‐PanNETs.

Abbreviations: AEs = adverse events, EUS = endoscopic ultrasound, F = functioning, MPD = main pancreatic duct, NF = nonfunctioning, NSAIDs = nonsteroidal anti‐inflammatory drugs, PanNET(s) = pancreatic neuroendocrine tumor(s), RFA = radiofrequency ablation.

### 5.1 Radiofrequency Ablation

EUS‐guided RFA is the most extensively studied ablative method for small, well‐differentiated PanNETs, especially insulinomas. Multiple prospective and retrospective series have reported that a single RFA session can achieve complete tumor ablation in 90%–95% of benign insulinomas under 2 cm in diameter [77, 78]. One key advantage of EUS‐RFA lies in its capacity to directly visualize the lesion and deliver thermal energy with minimal impact on surrounding tissues. Studies have described clinical success (i.e., normalization of blood glucose) in 95% of insulinoma patients, with markedly fewer complications than surgery [79, 80]. In NF‐PanNETs measuring up to 20 mm, EUS‐RFA has also demonstrated high rates of radiological response, with 80%–90% rates of complete ablation of the lesions [76, 81].

Despite encouraging short‐term data, questions remain regarding the long‐term oncological effectiveness of EUS‐RFA, particularly for NF‐PanNETs. Unlike surgery, which removes the tumor entirely and allows for lymph node dissection, thermal ablation does not provide pathological margin assessment or formal lymphadenectomy [82]. Ongoing trials (NCT03834701, NCT04520932) aim to refine inclusion criteria, especially for small NF‐PanNETs, expanding data on disease‐free survival and local recurrence. In a systematic review and meta‐analysis by Armellini et al., 20 studies, mostly retrospective series, including 183 patients with PanNETs treated with EUS‐RFA were included [83]. A total of 196 PanNETs (101 functioning and 95 nonfunctioning), ranging from 4.5 to 30 mm, were analyzed, with clinical effectiveness, defined as symptom resolution for F‐PanNETs and complete ablation for NF‐PanNETs, achieved in 95.1% (95% confidence interval [CI], 91.2%–98.9%) and 93.4% (95% CI, 88.4%–98.4%) of cases, respectively. Focusing on nonfunctioning, small (< 2 cm) lesions, the expected rate of complete tumor response ranges from 70% to 100%.

Overall complication rates for pancreatic EUS‐RFA range from 10% to 20% and are largely mild, including transient abdominal pain, low‐grade pancreatitis, or peripancreatic fluid collections [7]. Higher‐risk lesions, particularly those within 2 mm of the MPD, have shown pancreatitis rates as high as 40% [81]. Pancreatic duct stenosis is rare but recognized, whereas bleeding or infection of the puncture tract occurs infrequently; in fact, broad‐spectrum antibiotics are administered prophylactically to lower the risk of pancreatic or peripancreatic infection [84]. Some centers also give rectal nonsteroidal anti‐inflammatory drugs (NSAIDs) to reduce postprocedure risk of pancreatitis, although prophylaxis varies among institutions [85].

Conversely, in patients with insulinomas, a continuous 10% dextrose infusion prior to RFA and close glucose monitoring during and at least 24 h after the procedure are strongly recommended to avoid severe hypoglycemia caused by rapid tumor cell necrosis [86, 87].

Regarding NF‐PanNETs, in a retrospective study of 27 patients treated with RFA, 4 developed acute pancreatitis, and 3 of these subsequently formed a pancreatic fluid collection, requiring EUS‐guided drainage [88]. Thus, for minute NF‐PanNETs, which may have negligible progression risk, caution is advised when weighing the advantages of RFA against potential adverse events (AEs).

### 5.2 RFA Systems

One method for EUS‐RFA employs the Habib RFA probe (Habib EUS RFA, EMcision, London, United Kingdom), 1F monopolar electrode probe, inserted through a 19G or 22G EUS FNA needle and connected to a common electrosurgical generator. RF energy is delivered at 10–20 W in soft coagulation mode for 90–120 s per cycle [89]. A key advantage is compatibility with 22G needles, broadening its application. However, since it lacks integrated cooling, the probe can rapidly increase the tissue impedance, limiting the ablation volume. Currently, the Habib RFA probe is not available on the market [90].

An alternative is the EUSRA needle electrode (Taewoong Medical, Gimpo, Gyeonggi, South Korea), a 19G needle with an internal cooling mechanism and an uninsulated active tip (5–30 mm). Saline circulates via an external pump to prevent overheating, enabling longer energy delivery and a more uniform ablation zone. Connected to a proprietary RF generator (VIVA RF, Taewoong Medical, Gimpo, Gyeonggi, South Korea), power settings typically range from 30 to 50 W [91]. The procedure is halted when impedance spikes (~1000 Ω), indicating tissue charring. Cooling reduces the risk of overtreatment or undertreatment, ensuring predictable ablation.

With the monopolar system, the RFA probe is advanced through a prepositioned 19G/22G FNA needle. In contrast, the cooled system integrates the electrode tip within the 18G/19G needle. Once activated, the RF energy (30–50 W) induces echogenic microbubbles, forming a hyperechoic zone clearly visible on ultrasound.

### 5.3 Multiple Ablations Within a Single Session

For lesions larger than 15–20 mm, the needle may be repositioned to treat different tumor regions reproducing a sort of “fanning.” Some clinicians describe a “pullback” technique by placing the uninsulated tip in the deeper portion of the lesion and slowly withdrawing it to ablate along the track [92, 93]. Multiple passes can be performed in a single session if the ultrasound reveals any untreated residual tumor. Real‐time CE‐EUS can also highlight remaining perfused tumor areas for immediate reablation [94]. In some series, multiple EUS‐RFA sessions spaced days or weeks apart are used to fully treat large or multifocal neoplasms. Power settings vary among studies; most centers use 50 W for small (< 2 cm) PanNETs, with 10‐ to 20‐s pulses repeated once or twice. Others opt for 30 W delivered over 90–120 s to achieve a more uniform thermal distribution [81, 95].

### 5.4 Adverse Events and Oncological Limitations of EUS‐Guided RFA in PanNETs

Available data indicate that EUS‐guided RFA is feasible and generally safe in expert centers, but AEs are not rare, and the long‐term oncologic impact is still unclear. Across PanNET‐focused series, overall AE rates for pancreatic EUS‐RFA range from about 10% to 20%, with most events being mild and self‐limited, such as transient abdominal pain, low‐grade pancreatitis, and small peripancreatic fluid collections [7]. In the largest PanNET‐specific aggregation, 196 lesions (101 functioning and 95 nonfunctioning) in 183 patients underwent 326 RFA sessions, with 58 AEs (17.8% overall); severe AEs occurred in 3.1% of procedures and included six cases of severe pancreatitis, one infected abdominal collection, one MPD injury and two stenoses [83]. New‐onset diabetes was reported in approximately 0.3% of sessions in the same meta‐analysis.

Acute pancreatitis is the most frequent clinically relevant complication and accounts for roughly half of all AEs. In a meta‐analysis, pancreatitis occurred in about 6%–7% of sessions, whereas other single‐center series report rates up to 10%–15%, depending on lesion location and ablation protocol [83]. Risk is strongly influenced by the distance from the MPD: higher‐risk lesions, particularly those within 2 mm of the duct, have shown pancreatitis rates as high as 40% [83, 85]. Ductal complications are rare but clinically important; they can require endoscopic retrograde cholangiopancreatography (ERCP) with stenting and, in exceptional cases, surgery [83, 96]. These data support maintaining a safety margin of at least 2 mm from the MPD; lesions abutting the duct are generally poor candidates for EUS‐RFA outside of clinical trials, and in exceptional cases where ablation is still pursued, a prophylactic pancreatic duct stent may be considered in the preprocedural setting, bearing in mind that its protective effect is unproven and that ERCP itself carries a nonnegligible risk of pancreatitis.

Infectious and hemorrhagic events are less frequent but clinically significant, with peripancreatic or retroperitoneal fluid collections (approximately 3%–5%), sometimes infected, occasionally requiring percutaneous or EUS‐guided drainage [83, 88]. Overt bleeding, intramural gastric or splenic hematomas, and small‐bowel perforation are rare (< 1%–2%) and appear as isolated cases within the severe AE category [83, 96]. To reduce infectious risk, most centers administer prophylactic broad‐spectrum antibiotics before and after ablation [84], and many also use rectal NSAIDs to try to lower the risk of postprocedural pancreatitis, although consistent evidence of benefit is lacking, and local protocols remain heterogeneous [80, 85]. Prophylactic pancreatic stenting has been proposed for lesions close to the MPD, but ERCP itself carries a 3.5%–9.7% risk of post‐ERCP pancreatitis (up to about 15% in high‐risk patients), and available series have not shown that stenting can completely offset the risk associated with very short duct–tumor distance [85].

Functional PanNETs, particularly insulinomas, have additional safety issues related to abrupt hormonal changes. Rapid tumor necrosis can cause major shifts in insulin release and glycemic control. For this reason, in most insulinoma RFA series, a continuous 10% dextrose infusion is started before ablation and maintained during and for at least 24 h afterward, with close glucose monitoring to prevent severe hypoglycemia [97, 98].

For NF‐PanNETs, the risk–benefit balance is more complex. In a retrospective cohort of 27 patients with NF‐PanNETs treated with EUS‐RFA, four patients (about 15%) developed acute pancreatitis, and three of these evolved into pancreatic fluid collections requiring EUS‐guided drainage [88]. These data are consistent with the 10%–20% overall AE rate seen in larger PanNET series but become critical when considering minute NF‐PanNETs, which in surgical and surveillance cohorts often show very low progression risk under structured radiological follow‐up [76, 99, 100]. In these very small, incidentally discovered lesions, the morbidity of RFA, including the risk of pancreatitis and potential need for repeated interventions, may outweigh the expected natural history, and active surveillance remains a strong alternative.

The evidence base also has important structural limitations, as most PanNET ablation series are single‐center observational studies including fewer than 50 patients and using heterogeneous inclusion criteria for tumor size (4.5–30 mm), grade, functional status, and hereditary syndromes [80, 96].

Devices (Habib vs. EUSRA), power (30–50 W), application time (10–120 s), number of applications, and endpoints for terminating ablation (impedance rise, echogenic “cloud”) differ between centers [89, 95]. Definitions of “complete ablation” or “clinical success” vary, and histologic confirmation of complete necrosis is rare. Importantly, EUS‐RFA cannot provide margin status or lymph‐node clearance, so occult nodal or micrometastatic disease may persist even with apparently complete local responses.

Median follow‐up in RFA cohorts is usually 12–36 months, and few patients are followed beyond 3 years [77, 96]. For well‐differentiated PanNETs, which may relapse late even after radical surgery [76, 99], this is insufficient to define cure.

No randomized trials have compared EUS‐RFA with surgery or with active surveillance in sporadic small NF‐PanNETs, and robust data on progression‐free and overall survival, quality of life, and cost‐effectiveness are lacking. At present, EUS‐guided ablation for PanNETs should therefore be reserved for carefully selected patients, ideally in high‐volume centers and within multidisciplinary pathways, with explicit counseling that overall AE rates are around 15%–20%, severe events occur in about 3%, and long‐term oncologic outcomes remain uncertain [77, 101, 102]. Table 4 provides a general overview of relevant studies on EUS‐RFA for PanNETs.

**TABLE 4:** EUS‐guided radiofrequency ablation for PanNETs.

Author (year)	Study design	PanNETs	Lesion type; size	Technique details	Clinical success rate	Adverse events (AEs)	Follow‐up	Recurrence
Rizzatti et al. (2025) [103]	Prospective multicenter	60	F (insulinomas) and NF; ≤ 2 cm (F)/2.5 cm (NF)	EUSRA 19G cooled needle: power 30–50 W	97% (F‐PanNETs), 88% (NF‐PanNETs)	Mild AEs: 15%; Severe AEs: 1.7%	12 months	NR
Crinò et al. (2023) [80]	Retrospective multicenter	89	F (insulinomas) < 2 cm	EUSRA 18G–19G cooled needle; power 10–50 W	95%	Mild AEs: 18%; No severe AEs	23 months (median)	17%
Napoléon et al. (2023) [85]	Retrospective multicenter	64	F and NF G1, G2 and G3; < 3 cm	EUSRA 19G cooled needle, power 50 W	87% (F‐PanNETs), 72% (NF‐PanNETs)	Mild AEs: 11.2%; Severe AEs: 1.7%	13 months (median)	NR
Marx et al. (2022) [88]	Retrospective multicenter	27	NF G1 and G2 < 2 cm	EUSRA 19G cooled needle, power 30–50 W	93%	Mild AEs: 25.9% overall; No severe AEs	16 months (median)	7%
Barthet et al. (2019) [78]	Prospective multicenter	14	NF < 2 cm	18G cooled needle, ~50 W, one to two sessions	86% complete ablation at 1 year	AEs: 10%	12 months	14%

*Note:* Clinical success defined as complete tumor ablation on imaging for NF‐PanNETs or symptoms resolution for F‐PanNETs.

Abbreviations: AEs = adverse events, EUS = endoscopic ultrasound, F = functioning, G = grade, NF = nonfunctioning, NR = not reported in detail, PanNET(s) = pancreatic neuroendocrine tumor(s), W = Watt.

### 5.5 Alternative Ablation Techniques

#### 5.5.1 Ethanol Ablation

EUS‐EA involves injecting dehydrated alcohol directly into the tumor under EUS guidance, causing coagulative necrosis via tissue dehydration and vascular thrombosis [104, 105]. Ethanol is widely available and relatively inexpensive. Several studies demonstrate high technical feasibility for small or localized PanNETs [106, 107, 108]. However, ethanol distribution within the tumor can be uneven, raising concerns about partial treatment and higher recurrence rates [109, 110]. A recent multicenter, retrospective, propensity score–matched study compared EUS‐EA (97 patients) to surgical resection (188 patients) for small (< 2 cm) NF‐PanNETs [111]. The study reported that EUS‐EA had significantly fewer early major AEs (0% vs. 11.2%, *p* = 0.003) and a markedly shorter hospital stay (4 vs. 14.1 days, *p* < 0.001), with comparable 10‐year overall survival (97.1% vs. 90.7%) and disease‐specific survival (both 100%). Although complete ablation was achieved in 65% of EUS‐EA patients, nearly 46% of these experienced local recurrence (median, 34.5 months), whereas distant recurrence occurred in 2.7% of surgical patients; additionally, post–EUS‐EA pancreatitis was observed in 14.6% of sessions, despite the absence of early major AEs. Overall, the authors concluded that EUS‐EA preserves more pancreatic parenchyma and results in less endocrine dysfunction (33.3% vs. 48.6%) compared to surgery, suggesting that repeated ablations could be a possible strategy in selected cases [112, 113]. Table 5 summarizes relevant reports on EUS‐EA.

**TABLE 5:** EUS‐guided ethanol ablation for PanNETs.

Author (year)	Study design	PanNETs (n)	Lesion type; size	Technique details	Clinical success rate	Adverse events	Follow‐up	Recurrence rate
Matsumoto et al. (2025) [108]	Prospective multicenter	25	NF G1; ≤ 15 mm	EUS‐ethanol injection	88%	Mild AEs: 20%; severe AEs 4%	6 months	NR
So et al. (2023) [111]	Retrospective single‐center	97	NF G1 and G2; ≤ 2 cm	EUS‐ethanol injection (± lipiodol)	65%	Mild AEs: 14.6%; no severe AEs	NR	46% local recurrence
Choi et al. (2023) [114]	Retrospective single‐center	47	F and NF; NR	EUS‐ethanol injection	100% F‐PanNETs (NF‐PanNETs NR)	Mild to moderate AEs 32%; severe AEs 9%	18 months (median)	NR
Choi et al. (2018) [107]	Prospective single‐center	40	F (insulinomas) and NF G1 and G2; < 2 cm	EUS‐ethanol‐lipiodol injection	100% F‐PanNETs, 60% NF‐PanNETs	Mild AEs: 3%; no severe AEs	42 months (median)	No recurrence in ablated tumors or successfully treated F‐PanNETs

*Note:* Clinical success defined as complete tumor ablation on imaging for NF‐PanNETs or symptoms resolution for F‐PanNETs.

Abbreviations: AEs = adverse events, EUS = endoscopic ultrasound, F = functioning, G = grade, NF = nonfunctioning, NR = not reported in detail, PanNET(s) = pancreatic neuroendocrine tumor(s).

#### 5.5.2 Microwave Ablation

Microwave ablation delivers energy in the 900‐ to 2450‐MHz range to induce coagulative necrosis through frictional heating of water molecules within the tumor. Unlike ethanol, which relies on fluid diffusion, microwave ablation raises tissue temperature to approximately 80°C–90°C around the antenna tip. Small case series have demonstrated the feasibility of EUS‐guided microwave ablation in patients with insulinomas and NF‐PanNETs, with technical success achieved in 100% of treated cases, albeit a small patient sample [115, 116]. Complication rates ranged from 3% to 36%, primarily involving mild pancreatitis, fluid collections, or MPD strictures. Although promising, EUS‐guided microwave ablation remains relatively uncharted, with only limited data available [117]. Further questions persist about optimal power settings, ablation duration, needle size, and the number of sessions needed to reliably achieve complete necrosis. Given preliminary evidence suggesting favorable outcomes for microwave ablation in other solid tumors, future comparative studies are warranted to clarify whether it can offer more uniform intratumoral heating in fibrotic or densely stromal PanNETs.

## 6 Surgical Approach

Surgical resection remains the cornerstone of curative treatment for localized PanNETs, providing the highest likelihood of long‐term survival in patients with well‐differentiated, nonmetastatic disease [118]. The type of surgical procedure is tailored to tumor location: pancreaticoduodenectomy for lesions in the head, distal pancreatectomy for body and tail, and central pancreatectomy for selected small tumors to preserve pancreatic tissue. Enucleation may be considered for functioning tumors (particularly insulinomas) distant from the MPD, although it carries an increased risk of pancreatic fistula [119] Minimally invasive surgery, including laparoscopic and robotic techniques, is increasingly performed in high‐volume centers and has demonstrated noninferior oncological outcomes compared to open surgery, with reduced postoperative complications, shorter hospital stay, and improved recovery [120].

Routine lymphadenectomy is recommended, especially in nonfunctioning PanNETs ≥ 2 cm, due to the prognostic role of nodal metastases [121, 122].

All functioning PanNETs can be considered for surgical removal, regardless of their size [21]. Nonfunctioning PanNETs that are 2 cm or larger, or that cause symptoms, are also candidates for surgery [22]. The surgical indication for small (< 2 cm), incidentally discovered, nonfunctioning PanNETs remains debated [76, 99]. Although active surveillance is supported by some large retrospective and prospective studies [76, 123, 124], surgery is still favored in younger patients, in tumors showing growth or atypical imaging features, or in patients unwilling to undergo lifelong imaging follow‐up [125]. Grading, including the Ki‐67 value, is also an important factor in determining the surgical indication, in addition to other factors such as tumor size and symptomatology. Although the latest ENETS guidelines do not provide specific recommendations for resection based solely on preoperative grading [22], unlike the guidelines from the North American Neuroendocrine Tumor Society [37] and the European Society for Medical Oncology [37], grading still plays a significant role in prognostic assessment, therapeutic decision‐making, and the risk of postoperative recurrence [71, 126, 127, 128, 129, 130, 131]. In borderline resectable or locally advanced PanNETs, neoadjuvant therapy is emerging as a promising strategy to increase the rate of R0 resections and reduce intraoperative morbidity.

The phase II NEOLUPANET trial has recently investigated neoadjuvant PRRT (peptide receptor radionuclide therapy) with four cycles of [^177^Lu]‐Lu‐DOTATATE in patients with well‐differentiated, SSTR‐expressing PanNETs. Preliminary results indicate that this regimen induces radiological partial responses in 58% of cases and achieves significant tumor downstaging, enabling R0/R1 resection in 97% of patients without perioperative mortality, including lesions initially considered unresectable or borderline resectable. These findings support a “PRRT‐first” approach for downstaging borderline or locally advanced disease and refining surgical candidacy in this setting [76]. This approach may also help select patients with biologically indolent disease more likely to benefit from surgery. Surgical resection may also be indicated in metastatic disease for symptom control in functioning tumors or for debulking in highly selected cases, typically as part of a multidisciplinary treatment plan [21, 22].

## 7 Conclusions and Future Perspectives

EUS has evolved from a purely diagnostic procedure into a pivotal platform for both decision‐making and therapy in PanNETs. On the diagnostic side, its superior spatial resolution, contrast‐enhanced harmonic imaging, quantitative elastography, and n‐CLE allow morphologic and vascular characterization that surpasses cross‐sectional techniques, especially for lesions less than 20 mm.

Therapeutically, EUS‐RFA can induce high short‐term to midterm clinical and radiologic response rates in functional PanNETs ≤ 2 cm, but the supporting evidence is still largely based on small, mostly retrospective series with heterogeneous protocols and limited follow‐up, so that its true long‐term oncologic efficacy and safety profile compared with formal pancreatectomy remain uncertain. Similarly, EUS‐EA relies on nonrandomized cohorts with relevant rates of local recurrence, and both techniques should therefore be considered investigational, pending well‐designed prospective studies with prolonged follow‐up and, ideally, randomized trials directly comparing RFA and ethanol‐based ablation before they can be regarded as consolidated alternatives to surgery, even in frail or surgically ineligible patients. In parallel, the emerging experience with neoadjuvant [^177^Lu]‐Lu‐DOTATATE, as suggested by the phase II NEOLUPANET trial, highlights the potential of a PRRT‐first strategy to downstage borderline or locally advanced PanNETs and refine surgical candidacy, although its exact place in the therapeutic algorithm remains to be defined.

Emerging artificial intelligence and radiomics applications in EUS have been increasingly reported to not only differentiate PDAC from PanNETs [132, 133, 134], but also predict PanNET grading [135], providing additional prognostic information to guide management. In a multicenter convolutional neural network study analyzing more than 90,000 EUS frames from 378 patients, the model achieved 94% accuracy in distinguishing PDAC from PanNETs, including small or challenging lesions in chronic pancreatitis. These tools may enhance early detection, reduce interobserver variability, and support targeted tissue sampling, although prospective real‐time studies are still needed [136].

Combined ablation approaches that blend EUS‐RFA with microwave energy, intratumoral drugs, or thermosensitive nanoparticles seek to widen necrotic zones and potentiate immunogenic cell death. Systemic control could be boosted by next‐generation α‐emitters guided by ultrahigh‐resolution PET/CT, whereas multiomic analysis of core biopsies is expected to unveil predictive signatures and personalize the full therapeutic continuum from surveillance and ablation to surgery, PRRT, targeted agents, and immunotherapy.

The integration of advanced endoscopy, functional imaging, and molecular biology is driving an emerging approach that aims to deliver less invasive treatment while maintaining adequate oncologic control, preserving pancreatic function, and reducing treatment‐related morbidity. This strategy may improve long‐term outcomes and quality of life in patients with PanNETs, but requires confirmation in prospective, rigorously conducted studies.

## Funding

The authors have nothing to report.

## Data Availability

Data sharing not applicable to this article as no datasets were generated or analysed during the current study.
